# Clinical debriefing during the COVID-19 pandemic: hurdles and opportunities for healthcare teams

**DOI:** 10.1186/s41077-021-00182-0

**Published:** 2021-09-15

**Authors:** Jody L. Stafford, Esther Leon-Castelao, Albert J. Klein Ikkink, Sigrun A. Qvindesland, Munt Garcia-Font, Demian Szyld, Cristina Diaz-Navarro

**Affiliations:** 1 Department of Perfusion/Cardiothoracic Surgery, Cardiff and Vale University Health Board, Cardiff, UK; 2 Clinical Simulation Laboratory, Faculty of Medicine and Healthcare Sciences, University of Barcelona, Barcelona, Spain; 3 Wenckebach Simulation Center for Training, Education and Research, University Medical Center Groningen, Groningen, The Netherlands; 4 Department of Education, Stavanger University Hospital, Stavanger, Norway; 5 Center for Medical Simulation, Boston, MA USA; 6 Health Education and Improvement Wales, Cardiff, UK

**Keywords:** COVID-19, Healthcare teams, Clinical debriefing, Non-technical skills, Human factors, Safety

## Abstract

The COVID-19 pandemic and the subsequent pressures on healthcare staff and resources have exacerbated the need for clinical teams to reflect and learn from workplace experiences. Surges in critically ill patients, the impact of the disease on the workforce and long term adjustments in work and life have upturned our normality. Whilst this situation has generated a new ‘connectedness’ within healthcare workers, it also continues to test our resilience.

An international multi-professional collaboration has guided the identification of ongoing difficulties to effective communication and debriefing, as well as emerging opportunities to promote a culture of dialogue. This article outlines pandemic related barriers and new possibilities categorising them according to task management, teamwork, situational awareness and decision making. It describes their direct and indirect impact on clinical debriefing and signposts towards solutions to overcome challenges and, building on new bridges, advance team conversations that allow us to learn, improve and support each other.

This pandemic has brought clinical professionals together; nevertheless, it is essential to invest in further developing and supporting cohesive teams. Debriefing enables healthcare teams and educators to mitigate stress, build resilience and promote a culture of continuous learning and patient care improvement.

## Background

The COVID-19 pandemic continues to have a profound impact on healthcare workers, as it has posed unprecedented pressures and forced us all to adapt both professionally and personally to uncertainty and constant change during its peaks and troughs [1]. The need for healthcare institutions to support reflection and learning in the workplace is now more important than ever, yet rarely achieved [2]. The inclusion of clinical debriefing into routine safety behaviours offers an opportunity to reverse the status quo. This pandemic presents us with new hurdles to its implementation and delivery but also with new possibilities to further its use.

Debriefing is a conversation that promotes reflection and learning following an experience. In simulation education, it enables focussed discussions aiming to improve knowledge and performance [3]. Its incorporation into clinical practice allows the whole team to discuss real events, encourages reflection and allows a deep level of experiential learning. There is growing evidence that it contributes to improving clinical outcomes [4–6]. It contributes to building resilience, strengthening shared mental models and facilitating adaptation to changing circumstances, such as the ones faced in this crisis [7–9]. Clinical debriefing (CD) should encourage learning, patient safety and system improvement whilst providing psychological support to the whole team [10, 11].

COVID-19 has changed the way we live and work. Barriers to debriefing such as inadequate time, space and lack of standardisation, are well known [12]. Performance variability and continuous adaptation are at the core of complex ever-changing healthcare working environments [13], and markedly more so during the ongoing pandemic, which has prompted new ways of overcoming obstacles at individual, team and organisational levels, providing opportunities to address pre-existing and new challenges.

The Education Committee of the TALK Foundation (www.talkdebrief.org) is a working group composed by international debriefing experts that coordinate and support CD implementation initiatives across a wide range of cultures, as our network currently includes 18 countries. Our experiences during this pandemic have led to the identification of new dynamics which affect the performance of CD. A collaborative interprofessional and international discussion process involving our local teams, together with an awareness of the latest evidence, have allowed us to reach a broad and practical perspective on the current situation. This article focuses on our observations of how COVID-19 affects clinical behaviours, its direct and indirect impact on CD performance, and the openings that we have capitalised on during this time.

We highlight arising opportunities and provide recommendations aiming to optimise team debriefing, engaging quality improvement whilst providing a supportive culture, enabling teams to better overcome current pandemic challenges as well as to prepare for a potentially fraught recovery period.

## Challenges and new opportunities for clinical debriefing during a pandemic: considerations through the lens of a non-technical skills framework

The impact of COVID-19 on human factors is all encompassing, disrupting the way we behave, communicate, learn and reflect [1, 14]. Individuals and teams are at the core of healthcare systems and their abilities and limitations are key determinants of overall performance [15]. CD allows us to recognise how new conditions affect the way we work, take steps to support teams and improve together whilst fostering a healthcare culture of resilience and safety [8, 16].

In this article, we discuss how this pandemic has disrupted our performance across four categories: task management, team working, situation awareness and decision making. Building on our collective experience, we consider how this turmoil continues to affect our clinical practice as well as the subsequent debriefings, and share proposals for overcoming difficulties whilst making the most out of new opportunities. We have organised the issues identified, their implications for CD and our recommendations (Table 1) according to a non-technical skills (NTS) framework, as this taxonomy provides a befitting standardised approach to the observation and improvement of workplace behaviours both in clinical and simulation environments [17–20].

**Table 1:** COVID-related changes affecting clinical behaviour, their implications for CD, solutions and opportunities

COVID related changes affecting clinical behaviours	NTS category	Consequences/complexities affecting clinical debriefing	Solutions and new opportunities
Infection control measures prior to all clinical interactions	Task management	Potential patient care delay Emotional consequences, complex CD	Debrief supported by expert facilitators
Wearing PPE		Visual and auditory limitations Teams separate at the time of removing PPE (doffing)	Early planning of debriefing Pre-set regular debriefing Agree CD time during team briefing
Team distribution	Teamwork	Divided teams and isolated clinical areas, different COVID exposure	Virtual (online) debriefing
Staff shortages		Sickness, self-isolation, shielding Increased workload Lack of time Exhaustion	Gain institutional support to prioritise safety behaviours such as CD Engage staff by sharing the purpose and benefit of debriefing Short and structured debriefing
Redeployment		Increased critical care demand Reorganisation of services	Collaborate with debriefing experts Embed new safety practice (debriefing) Promote resilience
Leadership challenges		Shifting goals and guidelines, staff uncertainty	Identification of needs via debriefing Reinforcement of good practice
Limited direct contact with patient, relatives and staff	Situation awareness	Hindered information gathering Stress and frustration	Ensure psychological safety: supportive/empathetic debriefing
Changes to practice and environment		Rapid change, need to gain familiarity with new ways of working	Opportunity for meaningful interprofessional engagement with CD
Self-awareness		Reduced resilience Stress and exhaustion	Highlight wellbeing benefits of supportive debriefing
Changes to diagnostic and treatment options	Decision-making	Available/accessible resources Changes to local guidelines might generate self-doubt Risk v benefit during pandemic	Online multi-professional Debriefing to align decisions Share debriefing outcomes Consider the long term

### Task management

Task management refers to how we assign and organise activities in order to achieve our goals. It encompasses four elements: planning and preparation, prioritisation, maintenance of standards and utilisation of resources [18]. COVID-19 has mandated a profound change to the way we behave and perform routine duties in healthcare practice.

For instance, maintaining infection control standards has become an overarching priority, preceding patient care delivery. In areas with potentially infectious patients, such as emergency departments or acute admission wards, staff must wear comprehensive personal protective equipment (PPE) before initiating aerosol generating procedures (AGP), even if these are life-saving [21]. This means that if a patient in those locations requires cardiopulmonary resuscitation (CPR), all staff must focus on donning (putting on) PPE before providing active patient care. Delaying CPR due to infection control measures may challenge our core values and have an emotional impact which could make a subsequent debriefing complex.

In this case, as in difficult post-simulation debriefing [22], access to experienced facilitators with advanced training may be crucial to a successful learning conversation, considering the “dynamic balancing act” of providing a safe psychological environment [23]. In our exposure to debriefing multi-professional teams following critical interventions which have been delayed by necessary infection precautions, it has been essential to provide early support to team members, reassurance that the right priorities have been established and freedom for different team perspectives to become explicit in order to identify constructive and collaborative ways forward.

New tasks and resources introduced in order to maintain patient and staff safety include wearing visors and tight-fitting face masks or respirators, which are uncomfortable and hinder communication. In our experience, these visual and auditory barriers markedly affect information gathering and exchange during clinical processes and particularly during debriefing, leading to team members not hearing or misunderstanding each other. However, as team members doff (take it off) and change to lower protection PPE, they move away from the original clinical area and team. Ensuring that CD is carried out in these circumstances requires engaging the full team in early planning and identification of an appropriate time and place, and will benefit from organisational support [2, 13].

We propose that a regular time and place for debriefing conversations is scheduled whenever possible, as this promotes peer connection and wellbeing [16, 24]. For instance, some of our teams have established routine end of shift debriefing sessions prior to handover. Alternatively, the time for CD can be arranged during team briefing in areas such as operating theatres or critical care [24, 25].

### Teamwork

Teamwork alludes to collaborative task completion and team member satisfaction and includes coordination of activities, exchange of information, leadership and followership, identifying capabilities and mutual support. The demands of managing COVID-19 have led to a constant state of flux, including large-scale restructuring of the workforce, resulting in the disruption of team dynamics [1, 14].

Physical barriers and varying levels of PPE disrupt teamwork albeit increasing safety [26]. Ensuing challenges to communication, including in-situ debriefing, must be acknowledged. Additionally, team distribution has been altered, as clinical areas have been divided into zones determined by infection risk and proximity to AGP. This separates and isolates staff, hindering activity coordination, information exchange and CD. As COVID-19 has rapidly advanced the use of tele-communication, we recommend establishing virtual debriefing sessions, which help build team resilience [8].

Online debriefing eliminates social distancing concerns and is particularly useful when signposting to further learning resources; however, this conveys unique socio-cognitive challenges which require tailored strategies, for example expressing appreciation, promoting inclusivity and explicitly acknowledging team members, as described by Cheng and colleagues [27].

Staff shortages relating to sickness, post-exposure quarantine, post travel self-isolation or shielding from infection continue to compound our challenges. High pressures on elective and emergency services related to increasing patient complexity, delayed treatments and escalating waiting lists [28] may decrease the uptake of debriefing processes. However, these high pressures might make overworked and exhausted staff more likely to benefit from shared reflective practice [8, 9]. Gaining institutional support to ensure that safety behaviours are prioritised at times of crisis is paramount [2, 13]. Clinical debriefing leads should disseminate the benefits of debriefing for improvement, wellbeing and resilience [4–9, 17, 24] in order to encourage staff to engage with debriefing processes.

We suggest that CD sessions are brief and follow a structure familiar to the team such as TALK^©^ [29], DISCERN [30], INFO [31], TeamSTEPPS [32] or DISCOVER-PHASE [33].

In our case, we have implemented TALK^©^ for CD, as it is an easy, inclusive and scalable way to debrief, supported by short standardised training sessions (less than 2 h). This initiative has been actively supported at an institutional level and well received by multi-professional staff.

The disruption of routine services and staff reorganisation to accommodate the surge in critically ill patients, and further adaptations to accommodate increasing service demands, have disrupted healthcare silos and normal delivery of care [28]. This redirection has resulted in staff rapidly adapting to cope with emerging needs: initially, working groups such as ‘Proning Teams’ needed to be assembled to learn and perform unfamiliar tasks at a high standard [34]; more recently, clinical personnel have been mobilised to deliver vaccination programmes [35]. Whilst these shared purposes are largely embraced, arising challenges can be eased through co-learning during debriefing conversations. New collaboration opportunities may emerge as CD expertise becomes available through staff redistribution or changes to service delivery. We propose that redeployed experts such as simulation educators, wellbeing staff or psychologists are introduced to the principles of CD and use their pre-existing skills to facilitate team debriefing. As an example, educators have volunteered to support vaccination efforts, placing themselves in an ideal position to facilitate CD in these new teams. This experience might later allow the integration of CD training within simulation settings, which will in turn promote future workforce teams to share reflections through debriefing practice.

Rapid changes in local and national guidelines due to new knowledge, infection prevalence and resource prioritisation translate into shifting goals, staff uncertainty and leadership challenges. This provides an opportunity to promote debriefing at a healthcare management level by highlighting organisational advantages of CD: it offers the opportunity to involve all staff in identifying emerging needs and system strengths and weaknesses [13], as well as to promote a culture of safety whilst fostering staff resilience [8, 12]. For instance, routine debriefing following the introduction of new infection risk zones allows teams to deal with practical difficulties, engaging all staff in monitoring and adapting to this reconfiguration.

### Situation awareness

Situation awareness (SA) applies to overall awareness of patient care and the environment where it takes place [18], including any relevant aspects such as patient’s condition and evolution, team, resources and time as well as how we perceive relevant cues, interpret their meaning, build a mental model, and predict what could happen next [36]. Hence, SA incorporates the processes of gathering information, recognising and understanding and anticipating, which have been threatened by the extensive disruption caused by this pandemic, as it continues to affect team and individual performance [1, 28].

We have experienced restrictions to contact with patients, relatives and colleagues as a consequence of efforts to limit viral spread and to conserve PPE. For example, an anaesthetist working in a low infection risk area can only access higher risk zones after wearing comprehensive PPE and can only return to lower risk areas after showering and changing into clean clothing; as a consequence, visits across areas are minimised, which may lead to loss of early access to important preoperative information such as observing the patient’s anatomy to assess their airway.

Moreover, access to information is hindered by staff working in isolation, lack of straightforward patient-clinician interaction and minimal opportunities to communicate with relatives other than telephonically. Information gathering may be thwarted further by stress and tiredness. We should acknowledge that in this context, CD is likely to be affected by ongoing frustrations. A supportive and empathetic approach is essential in order to sustain psychological safety during debriefing [23].

Rapidly changing environments and practice pose an extraordinary demand on SA and may give rise to clinical errors. Gaining familiarity with new ways of working will require a concerted interprofessional effort [14], which may foster a renewed engagement with meaningful CD as an avenue to move forward cohesively [12]. For instance, the establishment of regular online debriefing has allowed teams to share new pathways, set up innovative information streams and regain a sense of community whilst adhering to social distancing rules, which in turn has reinforced the usefulness of CD.

Self-awareness decreases when faced with cognitive overload, stress and burnout; for teams and individuals to perform optimally during this protracted crisis, they must look after themselves and each other [14]. This also offers a fertile ground to promote debriefing, as it allows the identification of vulnerabilities and provides the opportunity for all staff and clinical leads to demonstrate solidarity and promote wellbeing [8, 24, 37].

### Decision-making

Decision-making involves selecting a course of action by identifying options, assessing risks and benefits, carrying out an appropriate action and re-evaluating. This balance has been hampered during this crisis by new priorities as well as different considerations in risk versus benefit analyses.

Diagnostic and treatment options for patients both with and without COVID-19 have been affected [28]. Resource availability and accessibility may promote conservative treatment strategies, reduce elective procedures and lead to demanding conversations with patients and colleagues. We recommend exploring virtual multi professional debriefing sessions to facilitate collective prioritisation and decision alignment [16, 27]. Sharing outcomes and lessons from these conversations may guide to shape planning and future strategies [2, 24].

## Conclusion

The COVID-19 pandemic has created many difficulties for healthcare teams over the last year which has impacted on debriefing processes. New challenges and barriers to communication have forced teams and individuals to adapt their practice.

This article applies the NTS taxonomy as a lens to consider some of the challenges of this pandemic and highlight their direct and indirect impact on CD. It also identifies possible solutions and opportunities based on the latest evidence which associates CD experiences during this crisis with increased team resilience and wellbeing. This new context should stimulate a renewed interest to embed clinical debriefing into everyday practice as a way of collaborative reflection and learning within individuals, teams and organisations. The recognition of local challenges through CD will guide system improvement and staff support measures over the foreseeable future.

The pandemic has brought clinical professionals together; it is essential to nurture and maintain these reinvigorated relationships across organisations and invest in further developing cohesive teams. Debriefing is a tool for healthcare teams and educators to provide emotional support, mitigate stress and ultimately promote a culture of continuous learning and improved patient care.

## Data Availability

Not applicable
